# Are there socioeconomic inequalities in cardiovascular risk factors in childhood, and are they mediated by adiposity? Findings from a prospective cohort study

**DOI:** 10.1038/ijo.2010.52

**Published:** 2010-03-16

**Authors:** L D Howe, B Galobardes, N Sattar, A D Hingorani, J Deanfield, A R Ness, G Davey-Smith, D A Lawlor

**Affiliations:** 1 Department of Social Medicine, University of Bristol, Bristol, UK; 2 Department of Social Medicine, MRC Centre for Causal Analyses in Translational Epidemiology, University of Bristol, Bristol, UK; 3 BHF Glasgow Cardiovascular Research Centre, Faculty of Medicine, University of Glasgow, Glasgow, Scotland, UK; 4 Epidemiology and Public Health, University College London, London, UK; 5 Institute of Child Health, University College London, London, UK; 6 Department of Oral and Dental Science, University of Bristol, Bristol, UK

**Keywords:** ALSPAC, child, inequality, cardiovascular

## Abstract

**Background:**

Socioeconomic gradients in adiposity were not present during childhood for previous generations, but have emerged in contemporary children. It is unknown whether this translates to socioeconomic gradients in associated cardiovascular risk factors in children, with consequent implications for inequalities in coronary heart disease (CHD) and diabetes when these children reach adulthood.

**Methods:**

Using data from 7772 participants aged 10-years from the Avon Longitudinal Study of Parents and Children, we examined the association between maternal education and a large number of cardiovascular risk factors (cholesterol, triglycerides, high-density lipoprotein, apolipoprotein, adiponectin, leptin, C-reactive protein (CRP), interleukin-6 (IL-6) and systolic and diastolic blood pressure), and examined whether inequalities were mediated by adiposity, measured by dual energy X-ray absorptiometry (DXA)-assessed total fat mass.

**Results:**

There were socioeconomic differences in a number of the cardiovascular risk factors (apolipoprotein B, systolic and diastolic blood pressure, CRP, leptin and IL-6). Inequalities were greater in girls than boys. Inequalities in CRP and leptin were completely mediated by adiposity. Inequalities in other cardiovascular risk factors were partially mediated by adiposity.

**Conclusion:**

This study showed important socioeconomic inequalities in adiposity and associated cardiovascular risk factors in a contemporary UK population of 10-year-old children. Differences between contemporary children and previous generations in the socioeconomic patterning of cardiovascular risk factors suggest future adults may have greater inequalities in diabetes and CHD than current adults. These findings highlight the importance of interventions aimed at preventing obesity in childhood, particularly among those of lower socioeconomic position.

## Background

Low socioeconomic position (SEP) in childhood is associated with higher risk of coronary heart disease (CHD)^1, 2, 3^ and diabetes^4, 5^ in later life, but the mechanisms underlying this are unclear. One possibility is that children from more deprived backgrounds are likely to be more obese and childhood obesity mediates the association of childhood SEP with CHD in adulthood. A systematic review of studies in contemporary populations of children from high-income countries found that most studies show socioeconomic differentials in adiposity, with those from more deprived socioeconomic backgrounds being more adipose.^6^ Furthermore, greater body mass index (BMI) and obesity in childhood from age 7 onwards to early adulthood have been shown to be associated with CHD risk in adulthood.^7^ However, it is unknown whether this association is due to tracking of BMI from childhood to adulthood or whether permanent changes in cardiovascular risk factors, such as lipid and blood pressure levels, occur in childhood as a result of greater adiposity. If the latter is true these adiposity-related risk factors changes might mediate the association of childhood SEP with adult CHD. As well as being associated with future risk of CHD, individuals from poorer socioeconomic backgrounds in childhood have been found to be more obese, more dyslipidaemic and more insulin resistant in adulthood than those from higher socioeconomic groups,^8, 9^ but few studies have been able to examine whether childhood SEP is associated with variation in vascular and metabolic traits in childhood, and if so to what extent these associations are mediated by adiposity.

One study in Denmark (*n*=933), Estonia (*n*=1103) and Portugal (*n*=1153) showed socioeconomic differentials in BMI, waist circumference, lipids (high-density lipoprotein (HDL), low-density lipoprotein and triglycerides) and circulating insulin.^10^ In Estonia and Portugal (relatively low-income countries), the socioeconomic differential in BMI was the opposite (lower BMI and waist circumference in those from poorer and less well-educated families) of that observed in Denmark, a high-income country, where findings were consistent with results from other high-income countries,^6^ that is, BMI and waist circumference were higher in those from poorer and less well-educated families. The socioeconomic gradients in metabolic markers were in the directions that would be anticipated given the observed relationships between SEP and BMI, that is, levels of low-density lipoprotein, triglycerides and circulating insulin were higher among those of lower SEP in Denmark and lower among those of lower SEP in Estonia and Portugal, and vice versa for HDL. Data from the 1992–2002 National Health and Nutrition Examination Survey from the United States showed that neither income nor education were associated with the metabolic syndrome in adolescents (based on having three or more extreme values for waist circumference, blood pressure, triglycerides, or fasting glucose, using national reference data).^11^ A review of studies published up to the year 2000 found little socioeconomic patterning of blood pressure, cholesterol, C-reactive protein (CRP), homocysteine or fibrinogen during childhood or early adulthood, but most studies were small.^12^ A study in Northern Ireland (*N*=509) found little socioeconomic difference in biological risk factors for CHD at ages 12 or 15, but did find differences in behaviours such as physical activity, diet and smoking, leading to the suggestion that socioeconomic differences in risky behaviours are likely to emerge earlier than differences in physiological factors.^13^

In this study, we analyse data from a research clinic of the Avon Longitudinal Study of Parents and Children (ALSPAC) held when the children were approximately 10-years old (mean age 9.9 years). A previous publication from this study showed that children from lower socioeconomic backgrounds tend to have higher total fat mass and greater BMI, but that there was no social gradient in lean mass.^14^ The investigators also showed that inequalities in adiposity were wider when using DXA-assessed total fat mass compared with BMI, and concluded that using BMI as a measure of adiposity in children may underestimate inequalities. We extend this earlier work by examining socioeconomic differentials in cardiovascular risk factors, and the extent to which these associations are mediated by adiposity measured by DXA-assessed total fat mass.

## Materials and methods

### Study population

ALSPAC is a prospective cohort study investigating the health and development of children. The full study methodology has been published elsewhere,^15^ and is also detailed on the study website (www.bristol.ac.uk/alspac). Briefly, pregnant women resident in one of three Bristol-based health districts with an expected date of delivery between 1 April 1991 and 31 December 1992 were invited to take part in the study. Of these women, 14 541 were recruited. From these pregnancies, there were 14 062 live-born children, 13 988 of whom were alive at 1 year. This study uses data from a clinic held at 10 years of age (clinics held between January 2001 and January 2003, mean age of children at clinic visits 9.9 years, s.d. 0.3), to which all surviving children with known contact details and whose parents had consented to continued participation were invited. A total of 7722 children attended the clinic, approximately 65% of those invited. Ethical approval for the study was obtained from the ALSPAC Law and Ethics Committee and the Local Research Ethics Committees.

### Measurements

A questionnaire at 32 weeks gestation asked mothers to report their educational attainment. Maternal educational attainment was categorised as below O-level (ordinary level; exams taken in different subjects usually at age 15–16 at the completion of legally required school attendance, equivalent to today's General Certificate of Secondary Education), O-level only, A-level (advanced-level; exams taken in different subjects usually at age 18), or university degree or above.

Puberty stage was assessed using questionnaires containing line drawings and questions based on stages described by Tanner,^16, 17^ which were completed within 1 year of attendance at the clinic at age 10; Tanner stage ranges between I and V, with I being least sexually mature.

All other variables used in this analysis were from the clinic at age 10. Adiposity was measured by DXA-assessed fat mass. A Lunar prodigy narrow fan beam densitometer was used to perform a whole body DXA scan in which bone content, lean and fat masses are measured. Total fat mass (kg) is used in these analyses. Height was measured to the last complete millimetre (mm) using the Harpenden Stadiometer. Blood pressure was measured using a Dinamap 9301 Vital Signs Monitor (Critikon, Tampa, FL, USA). Two readings of systolic and diastolic blood pressure were recorded and the mean of each has been calculated. Non-fasting blood samples were taken using standard procedures with samples immediately spun and frozen at −80 °C. The measurements were assayed in 2008 after a median of 7.5 years in storage with no previous freeze–thaw cycles during this period. Plasma lipids (total cholesterol, triglycerides and HDL-C) were performed by modification of the standard Lipid Research Clinics Protocol using enzymatic reagents for lipid determinations.^18^ Apo A1 and Apo B were measured by immunoturbidimetric assays (Roche UK, Welwyn Garden City, UK). Leptin was measured by an in house enzyme-linked immunosorbent assay validated against commercial methods.^19^ Adiponectin and high-sensitivity interleukin-6 (IL-6) were measured by enzyme-linked immunosorbent assay (R&D systems, Abingdon, UK) and CRP was measured by automated particle-enhanced immunoturbidimetric assay (Roche UK). All assay coefficients of variation were <5%. CRP, leptin, IL-6 and DXA-assessed total fat mass were all right-skewed, so log transformations were used in all analyses.

### Statistical analysis

The initial steps in our analyses were to quantify the associations between (i) maternal education and DXA-assessed total fat mass, (ii) maternal education and each of the cardiovascular risk factors and (iii) DXA-assessed total fat mass and each of the cardiovascular risk factors. Inequalities in (i) DXA-assessed total fat mass and (ii) each cardiovascular risk factor were summarised using the slope index of inequality (SII). The SII assumes an underlying continuous distribution of SEP; it is interpreted as the mean difference comparing the individual of lowest SEP with the individual of highest SEP on this hypothetical underlying continuous scale.^20^ When calculating the SII for DXA-assessed fat mass, we adjusted analyses for age, height and height squared. Adjustment for age was carried out in calculating the SIIs for cardiovascular risk factors. Gender differences in these inequalities were examined by testing for interactions. To examine whether any observed gender differences in adiposity inequalities were driven by differences in biological age between girls and boys, we tested for interactions with age and determined the level of adiposity inequality across tertiles of age. To quantify the associations between DXA-assessed total fat mass and each of the cardiovascular risk factors, we used linear regression adjusted for age, height and height squared. Again, we examined gender differences by testing for interactions.

We then examined the degree to which inequalities in the cardiovascular risk factors were mediated by adiposity. For one variable to mediate the association of an exposure with an outcome, it has to fulfil the following criteria: (i) be causally influenced by the exposure of interest, (ii) causally influence the outcome and (iii) attenuate the association of the exposure with the outcome.^21, 22^ For our specific question of whether adiposity mediated the association of SEP with cardiovascular risk factors, we are assuming that SEP is causally related to adiposity and that adiposity is causally related to cardiovascular risk factors. In prospective cohort studies in adults, there are robust associations between adiposity and cardiovascular risk factors in a wide range of populations, and in randomised controlled trials of interventions that effectively reduce weight these risk factors are reduced in ways predicted from observational studies.^23, 24^ In children there are fewer such trials but evidence here also suggests that weight reduction results in expected improvements in cardiovascular risk factors.^25, 26^ Furthermore, Mendelian randomisation studies, in which genetic variants that are robustly associated with adiposity throughout life are used as instrumental variables, support a causal effect of greater adiposity on adverse cardiovascular risk factors.^27, 28^ It is less easy to provide evidence that SEP causally affects childhood adiposity, although the association is consistently observed across most high-income countries.^6^

Traditionally, mediation has been assessed by standard regression techniques; mediation by adiposity is assumed to be present if the association between SEP and a cardiovascular risk factor is attenuated to the null after adjustment for adiposity.^29^ In our analyses, we used path analysis^30^ to examine mediation of the inequalities in cardiovascular risk factors by adiposity. Path analysis is preferable to standard regression techniques because it allows estimation of the total effect of maternal education on each cardiovascular risk factor (equivalent to the crude, unadjusted association) and the indirect effect of maternal education (that is, the effect mediated by adiposity), with confidence intervals around each of these estimates.^31^ In standard regression methods, the ‘adjusted' effect estimate is a measure of the association between SEP and the cardiovascular risk factor holding adiposity constant. In contrast, the indirect effect estimate from path analysis holds SEP constant. Indirect effects therefore reflect the difference in cardiovascular risk factor that would be observed if the whole population moved from the adiposity level of the highest SEP group to the adiposity level of the lowest SEP group.

We present standardised linear regression coefficients for both the total and indirect effects of maternal education on each cardiovascular risk factor, that is, each coefficient for total effects represents the s.d. change in the cardiovascular risk factor for a one s.d. increase in maternal education. The size of these standardised total and indirect coefficients can then be compared with estimate the proportion of the SEP—cardiovascular association that is mediated by adiposity. For example, if the total coefficient was 1 and the indirect coefficient was 0.5, we could estimate that half of the effect of maternal education on the cardiovascular risk factor was operating through adiposity. Analyses were adjusted for age, height and height squared.

Path analyses were conducted in MPlus version 5.21 (Muthén and Muthén, Los Angeles, CA, USA),^32^ other analyses were conducted using Stata version 10.1 (Stata Corporation, Texas 2008, College Station, TX, USA).

### Dealing with missing data

Of the 7722 children attending the clinic, 8.8% had missing data for maternal education and 5.0% for DXA-assessed total fat mass. Levels of missing data were 1.5% for blood pressure. Missing data levels are higher for the blood assay, 34.2%, because some parents and children did not consent to the blood test.

Missing data levels were higher among children who were female, older, more adipose, and who had lower maternal and paternal education, lower parity mothers, lower birth weight and length, lower maternal age, lower gestational age at delivery, shorter and heavier mothers. We used multivariate multiple imputation to impute missing variables, including all covariables and potential predictors in the imputation equations. We used switching regression in Stata as described by Royston.^33^ We carried out 20 cycles of regression switching and generated 10 imputation data sets. The multiple multivariate imputation approach creates a number of copies of the data (in this case we generated 10 copies) each of which has imputed values for those that are missing, with an appropriate level of randomness, by chained equations.^33^ The main analysis results are obtained by averaging across the results from each of these 10 data sets using Rubin's rules and the procedure takes account of uncertainty in the imputation as well as uncertainty because of random variation.^33^ We imputed data for those with missing data who attended the 10-year follow-up clinic. Hence, the analyses based on these multivariate imputations all include data from 7722 participants.

We repeated all analyses including only those with complete data (without imputation). Multiple imputation resulted in some strengthening of associations between SEP and both adiposity and vascular and metabolic markers, because those with missing data are more likely to be of lower SEP and more adipose. Differences between analysis of the non-missing data set and the imputed data sets were, however, small. Multiple imputation results are presented in this paper, and non-missing data set analysis is available in web tables.

### Additional indicators of SEP and adiposity

We repeated analyses using paternal education and household social class as indicators of SEP. Results were very similar to those presented here for maternal education and so are not presented or discussed further (available on request from investigators). We also repeated analyses using BMI and waist circumference as measures of adiposity. Consistent with findings from a previous study using these data,^14^ observed associations between adiposity and both SEP and the cardiovascular risk factors were somewhat stronger when DXA-assessed fat mass was used compared with BMI. Results using waist circumference as the adiposity measure tended to be intermediate to those using BMI and DXA-assessed fat mass (available on request from investigators).

## Results

Children attending the 10-year follow-up clinic tended to have better educated mothers, older mothers and higher birth weights compared with the full cohort, but no differences in maternal pre-pregnancy BMI were observed (Supplementary table 1).

The socioeconomic profile of clinic participants and the mean levels of DXA-assessed fat mass and each of the cardiovascular risk factors are presented in Table 1. Girls tended to be more adipose than boys and have higher levels of the cardiovascular risk factors, for example, mean CRP levels were 0.22 mg l^–1^ in boys and 0.34 mg l^–1^ in girls. Information on pubertal status was available in 88% of children attending the research clinic. Most were at an early Tanner stage (82% stages I or II; 96% stages I–III) and so it is unlikely that associations will have been affected by puberty.

### Association of maternal education with adiposity

The relationship between DXA-assessed total fat mass and SEP differed between boys and girls (*P*-value for interaction 0.0052). All analyses are therefore presented separately for boys and girls.

Both boys and girls showed socioeconomic differentials in DXA-assessed total fat mass, with children from lower SEP households being more adipose (Table 2 and Supplementary table 2 for analysis of non-missing data set). Inequalities were wider in girls compared with boys.

**Figure 1:**
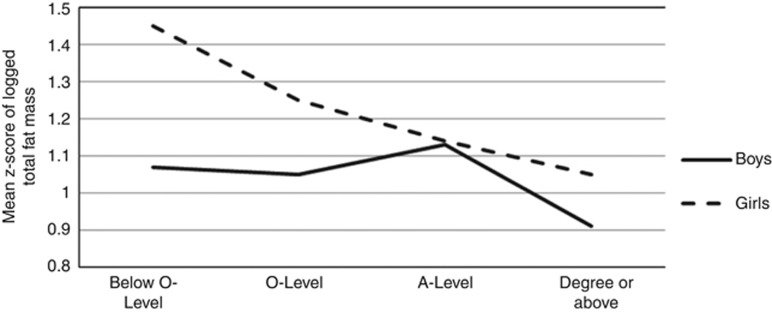
Mean levels of *z*-scores total fat mass across categories of maternal education for boys and girls.

When we looked at associations by categories of the SEP indicators (as opposed to calculating the SII as a summary measure) it appeared that not all associations were linear across the distribution (Figure 1). Among girls, there was a downward trend in DXA-assessed total fat mass across increasing categories of maternal education. In boys, however, there appeared to be more of a threshold effect, socioeconomic differences in DXA-assessed total fat mass were largely driven by lower levels among children whose mothers hold a degree and little socioeconomic gradient across the lower categories of maternal education (mean levels of DXA-assessed fat mass across categories of maternal education are presented in Supplementary table 3).

**Figure 2:**
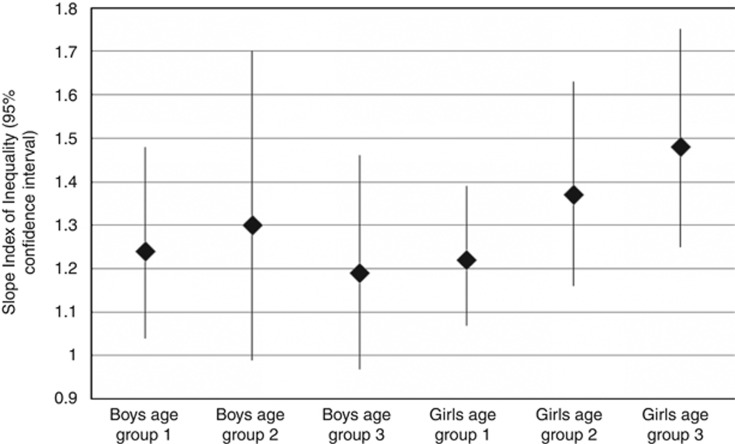
Slope index of inequality for DXA-assessed total fat mass, using maternal education as the SEP indicator, separately by tertiles of age and gender. Age ranges of tertiles were boys: 105–118 months, 118–120 months, 120–140 months, and girls: 106–118 months, 118–120 months, 120–140 months.

To examine whether the difference in adiposity inequalities between boys and girls was driven by the girls being biologically older (that is, closer to puberty), we determined how the inequalities changed with increasing age. Within both boys and girls, there was evidence of a gradient in inequality across age (*P*-value for interaction between age and maternal education <0.001 for both boys and girls). When the analyses are stratified by tertiles of age, however, the trend of widening inequality with increasing age was observed clearly within girls but not within boys (Figure 2).

### Association of maternal education with cardiovascular risk factors

Evidence for gender differences in the relationship between SEP and systolic and diastolic blood pressure was not strong (*P*-values for gender interactions 0.13. and 0.02). There was strong evidence of gender differences in the associations of maternal education with all other risk factors (*P*-values for interactions all ⩽0.001). All results were therefore presented separately for boys and girls, including blood pressure analyses for consistency.

As with adiposity, associations between SEP and cardiovascular risk factors tended to be stronger among girls than boys. For both boys and girls, weak associations were observed between maternal education and CRP, IL-6 and leptin, and stronger associations were observed for Apo B, systolic and diastolic blood pressure (diastolic blood pressure girls only). Children of less educated mothers had higher values of each of these risk factors (Table 2, Supplementary table 2 for analysis of non-missing data set, and Supplementary table 4 for mean levels across categories of maternal education). There was little evidence of socioeconomic differentials in the other cardiovascular risk factors (cholesterol, triglycerides, HDL, Apo A1 and adiponectin).

### Association of adiposity with cardiovascular risk factors

There was evidence of gender differences in the associations of some but not all cardiovascular risk factors with DXA-assessed total fat mass (for example, *P*-values for interactions 0.003 for triglycerides, <0.001 for adiponectin, <0.001 for leptin, 0.41 for systolic blood pressure and 0.27 for IL-6). However, for clarity and consistency all results are presented separately for boys and girls.

There was evidence for an association between DXA-assessed total fat mass and each of the cardiovascular risk factors (Table 3, and Supplementary table 5 for analysis of non-missing data set). An increase in total fat mass was associated with a decrease in HDL, Apo B and adiponectin, and an increase in each of the other cardiovascular risk factors.

### Mediation of socioeconomic inequalities in cardiovascular risk factors by adiposity

The inequalities in leptin and CRP were almost completely mediated by adiposity, because the coefficients of the total and indirect pathways are approximately the same size (Table 4). The degree to which inequalities in the other cardiovascular risk factors were mediated by adiposity was variable. For Apo B and IL-6, the coefficients for the indirect pathway (that is, mediated by adiposity) were approximately half to a third of the total effect size. For blood pressure, a lower proportion of the socioeconomic effect appears to be mediated by adiposity—the size of the coefficient for the indirect pathways is approximately half to a quarter of the total effect size in girls and a fifth to a sixth in boys.

Although no association was observed between maternal education and cholesterol, triglycerides, HDL, Apo A1 or adiponectin, there is evidence of an indirect pathway between maternal education and each of these outcomes through total fat mass. This is because each of these outcomes is associated with total fat mass, which itself is associated with maternal education.

## Discussion

### Inequalities in cardiovascular risk factors, and mediation by adiposity

Inequalities in some, although not all, cardiovascular risk factors were evident among these young children, with socioeconomic differentials generally being wider in girls than boys. For both boys and girls, weak associations were observed between maternal education and CRP, IL-6 and leptin, and stronger associations were observed for Apo B, systolic and diastolic blood pressure (inequalities in diastolic blood pressure were present only in girls).

The extent to which the inequalities in these cardiovascular risk factors were mediated by adiposity was variable. Almost the entire association between maternal education and leptin and CRP was mediated through fat mass. This is an expected result for leptin, given its very strong correlation with fat mass. For IL-6, Apo B, systolic and diastolic blood pressure there was evidence of mediation through adiposity, but the proportion of the inequalities mediated through adiposity was lower.

Although a gradient of higher levels of adult obesity with decreasing SEP has long been established in high-income countries,^34^ a similar socioeconomic gradient in childhood adiposity is a relatively recent phenomenon. In the past, disadvantaged groups would have been more likely to be less adipose rather than suffering from a higher burden of obesity. A review of cohorts published in 2002 (children aged up to 18 and young adults aged up to 24, who had largely been born between the 1940s and 1980s, although some studies of children born in the early 1990s were included) did not show socioeconomic inequalities in adiposity in childhood, but did show some evidence of socioeconomic patterning of adiposity in young adults.^12^ Other cardiovascular risk factors, including two that showed a socioeconomic gradient in this study (blood pressure and CRP), were also not found to be socially patterned in children and younger adults in previous generations.^12^ In a study of Glasgow students (attending the University Health Service between 1948 and 1968), lower childhood parental occupational social class did not predict early adult adiposity (mean age 23 years in men and 20 years in women), but did predict later adult BMI (mean age 39 years in men and 36 years in women), in spite of the little heterogeneity in their adult SEP.^35^ This lack of social gradient in early life adiposity among cohorts of today's adults, together with the fact that relatively few individuals would have been overweight or obese in childhood in these cohorts, implies that childhood obesity may not be a major contributor to the socioeconomic gradients in adult cardiovascular disease observed in many studies of contemporary adults who were born during the 1940s or earlier.^36^ However, the socioeconomic patterning of adiposity and other cardio;hyphen-qj;vascular risk factors we observe in these contemporary children suggests that socioeconomic gradients in adult adiposity, and also therefore in cardiovascular disease and particularly diabetes, may widen as today's children become adults. Interventions targeting inequalities in childhood adiposity may therefore be an important aspect of prevention of widening diabetes and cardiovascular disease inequalities in the future when these children become adults.

As with adiposity, blood pressure tends to be higher among lower socioeconomic groups in current adults.^37, 38^ Social patterning of blood pressure was not observed in children among previous generations.^12, 39^ We did see a socioeconomic gradient in blood pressure in the children in this study, implying that inequalities in hypertension may widen as these children become adults. It has been shown in this population that blood pressure is associated with both fat and lean mass.^40^ For diastolic blood pressure in both boys and girls, and additionally for systolic blood pressure in boys, a large proportion of the association with SEP in our analyses was not mediated by adiposity. This suggests that factors other than adiposity are contributing to socioeconomic inequalities in blood pressure, even in these young children. These factors could possibly include dietary patterns, physical activity or other potentially modifiable risk factors for high blood pressure, but require further exploration.

ApoB was more strongly socially patterned in this population than plasma cholesterol. Previous research has suggested that ApoB is more strongly related to insulin resistance than cholesterol and related risk factors (for example, triglycerides, CRP, and so on).^41^ It has also been shown to be potentially more predictive of fatal myocardial infarction than cholesterol.^42^ The association with ApoB, as with other risk factors was, however, largely mediated through socioeconomic inequalities in adiposity.

### Gender differences in inequalities in adiposity

We observed socioeconomic inequality in adiposity in both boys and girls, but the disparities were wider among girls. Among girls, there was a clear gradient of decreasing total fat mass across all categories of increasing maternal education. Among boys, in contrast, adiposity was lower among boys whose mothers had the highest educational level, but similar across the lower categories of maternal education.

The association between SEP and adiposity is known to be generally stronger in adult women than men,^34^ but gender differences are less consistent in contemporary children. In a systematic review of socioeconomic status and adiposity in school-aged children using studies from Western developed countries published since 1989, over half of the 19 studies reporting associations separately for boys and girls showed no gender difference in the association. Findings from the remaining studies were inconsistent.^6^ Contemporary children may be shifting towards the social patterning observed in adults, implying again that inequalities may be widening over time and with this becoming more prominent in females. Gender differences in the social patterning of adiposity are largely unexplained. It may, at least in part, be because of gender differences in the social patterning of physical activity. There is some evidence that boys are more likely to participate in sport than girls, regardless of their SEP^43^ and that the inverse association between physical activity and adiposity tends to be stronger and more common among boys than girls.^44, 45^ Within ALSPAC, there is little evidence of a socioeconomic gradient in physical activity,^46^ but there is evidence that boys are more likely to participate in moderate to vigorous activity,^46^ which has been shown to be more important than total activity for preventing obesity.^45^ Furthermore, women of higher SEP may be more susceptible to pressure to stay slim, and children may be becoming exposed to such pressures at this early age.

It is possible, however, that the weaker associations between SEP and adiposity and cardiovascular risk factors observed in boys in our study simply reflects girls at this age being biologically older than boys, that is, closer to puberty. The vast majority of both boys and girls were pre-pubertal in this cohort according to their Tanner stage (Table 1), but this relatively crude measure cannot give a specific indication of biological age. The hypothesis that the gender differences observed in this study may be at least partially driven by girls being biologically older is supported by the fact that we saw clear age-gradients in the social patterning of obesity within girls, with older girls showing greater socioeconomic differentials than younger girls, but less clear age patterning of inequality among boys.

### Study strengths and limitations

A major strength of this study is that it is the largest study to date to report socioeconomic inequalities in a wide range of cardiovascular risk factors in a cohort of contemporary children, and to examine the extent to which these inequalities are mediated by adiposity using DXA-assessed total fat mass.

We used path analysis to determine mediation of inequalities in cardiovascular risk factors by adiposity. This permits estimation of the proportion of the effect of SEP that is mediated through adiposity. We adjusted analyses for potential confounders (age, height and height squared) but the interpretation of the results from path analysis as causal estimates rests on the assumption of no unmeasured or residual confounding, similar to the use of standard regression methods.

Participation in most research studies is socially patterned, and ALSPAC is no exception. Those attending the follow-up clinic tended to be of higher SEP than the full cohort. This may have resulted in an underestimation of the inequalities in cardiovascular risk factors. There was minimal missing data among clinic participants for most variables but approximately one-third had data missing on blood assay risk factors. However, the results based on analysis of the non-missing data set and analyses using multivariate multiple imputation were similar and the similar patterning of associations with adiposity (for which there was very little missing data) and blood assay results, suggest that missing data has not resulted in major bias.

Offspring blood tests were completed on non-fasting blood samples but the majority of measures are not appreciably altered by this approach.^47, 48, 49^ Non-fasting triglyceride is at least as strongly linked to vascular outcomes as is fasting triglyceride,^50^ suggesting that for triglycerides also fasting status may not result in important biases with associations.

### Implications

Our work shows important socioeconomic inequalities in adiposity and associated cardiovascular risk factors, particularly in girls, in a contemporary UK population of 10-year-old children. Such childhood associations were not present in previous generations. Our results suggest that inequalities in adiposity, cardiovascular disease and diabetes may widen over time as these cohorts of children become adults. These findings highlight the importance of interventions aimed at preventing obesity in childhood. Population-based interventions, if successful, may have a greater overall population effect on the reduction of obesity and its associated complications than interventions targeted at lower socioeconomic groups. However, it is well known that many interventions are initially taken up to a greater extent by more socially advantaged groups, leading to a widening of socioeconomic differentials. To reduce socioeconomic inequalities in obesity, cardiovascular disease and diabetes, it will be necessary to either target interventions at disadvantaged groups, or to attempt to promote their participation in population-wide interventions.

## Figures and Tables

**Table 1:** Characteristics of study participants

*Parental socioeconomic characteristics*^a^				
			N (%)	
Maternal education	Less than O-level		1577 (22.4)	
	O-level		2476 (35.2)	
	A-level		1875 (26.6)	
	Degree or above		1117 (15.9)	
				
*Offspring adiposity*				
	*Boys*		*Girls*	
	N	*Mean (95% CI)*	N	*Mean (95% CI)*
Total fat mass (kg)^b^	3617	6128.88 (6009.95–6250.17)	3716	8558.06 (8418.56–8699.87)
				
*Offspring levels of cardiovascular risk factor*				
	*Boys*		*Girls*	
	N	*Mean (95% CI)*	N	*Mean (95% CI)*
Cholesterol (mmol l^–1^)	2575	4.20 (4.18–4.23)	2511	4.34 (4.31–4.37)
Triglycerides (mmol l^–1^)	2575	1.11 (1.09–1.13)	2511	1.17 (1.15–1.19)
High-density lipoprotein (mmol l^–1^)	2575	1.44 (1.42–1.45)	2511	1.37 (1.35–1.38)
Apolipoprotein A1 (mg per 100 ml)	2575	138.40 (137.61–139.18)	2511	133.70 (132.93–134.47)
Apolipoprotein B (mg per 100 ml)	2573	57.00 (56.50–57.47)	2511	61.49 (60.96–62.02)
Adiponectin (mg ml^–1^)	2575	12.74 (12.53–12.94)	2510	13.53 (13.31–13.75)
C-reactive protein (mg l^–1^)^b^	2575	0.22 (0.21–0.23)	2511	0.34 (0.33–0.36)
Leptin (ng ml^–1^)^b^	2575	4.77 (4.64–4.92)	2509	7.58 (7.36–7.80)
Interleukin-6 (pg ml^–1^)^b^	2567	0.77 (0.74–0.79)	2509	0.96 (0.93–0.99)
Systolic blood pressure (mm Hg)	3750	102.52 (102.23–102.81)	3857	103.09 (102.78–103.39)
Diastolic blood pressure (mm Hg)	3750	57.19 (56.98–57.39)	3860	57.75 (57.55–57.95)
				
*Pubertal stage assessed by Tanner questionnaires*				
	*Boys*		*Girls*	
	*Prevalence*, n/N (%)		*Prevalence*, n/N (%)	
I	1300/3223 (40.3)		2458/3555 (69.1)	
II	998/3223 (31.0)		798/3555 (22.5)	
III	704/3223 (21.8)		242/3555 (6.8)	
IV	215/3223 (6.7)		56/3555 (1.6)	
V	6/3223 (0.2)		1/3555 (0.03)	

Abbreviation: CI, confidence interval.

a Distributions do not differ by gender and are presented for male and female offspring combined.

b Geometric means.

**Table 2:** Inequalities in DXA-assessed total fat mass and cardiovascular risk factors at 9.9 years, quantified by the SII by maternal education.

	*Boys,* N*=3809*		*Girls,* N*=3913*	
	*SII*	*(95% CI)*	*SII*	*(95% CI)*
*Inequalities in adiposity*				
*difference in geometric means comparing most to least deprived (null value=1)*				
Total fat mass^a^	1.21	(1.08 to 1.36)	1.34	(1.23 to 1.46)
				
*Inequalities in cardiovascular risk factors*				
*difference in means comparing most to least deprived (null value=0)*				
Cholesterol (mmol l^–1^)	0.094	(−0.0059 to 0.19)	0.098	(−0.017 to 0.21)
Triglycerides (mmol l^–1^)	0.0016	(−0.088 to 0.091)	0.011	(−0.092 to 0.11)
High-density lipoprotein (mmol l^–1^)	0.017	(−0.027 to 0.061)	−0.021	(−0.066 to 0.024)
Apolipoprotein A1 (mg per 100 ml)	2.20	(−0.66 to 5.05)	0.60	(−2.50 to 3.70)
Apolipoprotein B (mg per 100 ml)	2.57	(0.88 to 4.26)	3.53	(1.55 to 5.52)
Adiponectin (mg ml^–1^)	0.41	(−0.34 to 1.16)	−0.44	(−1.35 to 0.47)
Systolic blood pressure	2.63	(1.52 to 3.73)	2.82	(1.69 to 3.94)
Diastolic blood pressure	1.25	(0.45 to 2.04)	1.73	(0.97 to 2.49)
				
*difference in geometric means comparing most to least deprived (null value=1)*				
C-reactive protein (mg l^–1^)	1.27	(1.03 to 1.57)	1.43	(1.20 to 1.70)
Leptin (ng ml^–1^)	1.10	(0.98 to 1.34)	1.25	(1.10 to 1.41)
Interleukin-6 (pg ml^–1^)	1.16	(1.06 to 1.33)	1.10	(0.96 to 1.27)

Abbreviations: CI, confidence interval; SII, slope index of inequality.

a Additionally adjusted for height and height squared.

*N*=7722 participants with multivariate imputation.

All analyses are adjusted for age.

SII represents the mean (or geometric mean) difference between the individuals of lowest and highest socioeconomic position on the hypothetical underlying continuous distribution of maternal education.

**Table 3:** Association between DXA-assessed total fat mass and cardiovascular risk factors at age 9.9 years

	*Boys,* N*=3809*		*Girls,* N*=3913*	
	*β*	*95% CI*	*β*	*(95% CI)*
Cholesterol (mmol l^–1^)	0.00002	(0.00001 to 0.00002)	0.00002	(0.00001 to 0.00002)
Triglycerides (mmol l^–1^)	0.00003	(0.00002 to 0.00003)	0.00003	(0.00002 to 0.00003)
High-density lipoprotein (mmol l^–1^)	−0.00002	(−0.00002 to −0.00001)	−0.00002	(−0.00002 to −0.00002)
Apolipoprotein A1 (mg per 100 ml)	−0.0006	(−0.0008 to −0.0004)	−0.0007	(−0.0009 to −0.0005)
Apolipoprotein B (mg per 100 ml)	0.0006	(0.0005 to 0.0007)	0.0007	(0.0005 to 0.0008)
Adiponectin (mg ml^–1^)	−0.09	(−0.14 to −0.05)	−0.17	(−0.21 to −0.13)
Systolic blood pressure	0.0007	(0.0006 to 0.0007)	0.0007	(0.0006 to 0.0008)
Diastolic blood pressure	0.0003	(0.0002 to 0.0003)	0.0003	(0.0002 to 0.0003)
Logged C-reactive protein (mg l^–1^)	0.0001	(0.00009 to 0.0001)	0.0001	(0.0001 to 0.0001)
Logged leptin (ng ml^–1^)	0.0001	(0.0001 to 0.00013)	0.0001	(0.0001 to 0.0001)
Logged interleukin-6 (pg ml^–1^)	0.00004	(0.00002 to 0.00005)	0.00004	(0.00003 to 0.00005)

Abbreviation: CI, confidence interval.

*N*=7722 participants with multivariate imputation.

Linear regression coefficients from regressions of total fat mass on the cardiovascular risk factor, adjusted for age, height and height squared. Age, height and height squared were centred on mean values (for boys and girls separately), such that coefficients represent the change in the cardiovascular risk factor for a 1 kg increase in total fat mass at 9.9 years in a child of average height.

**Table 4:** Mediation of the association between maternal education and cardiovascular risk factors by DXA-assessed total fat mass: standardised direct and indirect effects of maternal education

*Cardiovascular risk factor*	*Boys*, N*=3809*		*Girls*, N*=3913*	
	*Total effect (95% CI)*^a^	*Indirect effect (95% CI)*^b^	*Total effect (95% CI)*^a^	*Indirect effect (95% CI)*^b^
Cholesterol (mmol l^–1^)	0.030 (−0.10 to 0.070)	0.009 (0.004 to 0.015)	0.030 (−0.012 to 0.073)	0.014 (0.008 to 0.020)
Triglycerides (mmol l^–1^)	0.004 (−0.036 to 0.044)	0.011 (0.005 to 0.017)	0.014 (−0.027 to 0.055)	0.026 (0.017 to 0.034)
High-density lipoprotein (mmol l^–1^)	0.012 (−0.028 to 0.052)	−0.014 (−0.022 to −0.007)	−0.031 (−0.071 to 0.009)	−0.030 (−0.039 to −0.020)
Apolipoprotein A1 (mg per 100 ml)	0.033 (−0.007 to 0.073)	−0.007 (−0.011 to −0.002)	−0.001 (−0.041 to 0.039)	−0.018 (−0.025 to −0.011)
Apolipoprotein B (mg per 100 ml)	0.042 (0.002 to 0.082)	0.015 (0.007 to 0.023)	0.063 (0.022 to 0.10)	0.025 (0.016 to 0.033)
Adiponectin (mg ml^–1^)	0.022 (−0.018 to 0.062)	−0.001 (−0.004 to 0.002)	−0.022 (−0.062 to 0.019)	−0.016 (−0.023 to −0.010)
Systolic blood pressure	0.10 (0.068 to 0.13)	0.021 (0.010 to 0.031)	0.095 (0.064 to 0.13)	0.037 (0.026 to 0.049)
Diastolic blood pressure	0.069 (0.036 to 0.10)	0.012 (0.006 to 0.019)	0.083 (0.050 to 0.12)	0.022 (0.015 to 0.029)
Logged C-reactive protein (mg l^–1^)^a^	0.031 (−0.008 to 0.070)	0.028 (0.014 to 0.041)	0.063 (0.024 to 0.10)	0.052 (0.037 to 0.068)
Logged leptin (ng ml^–1^)^a^	0.047 (0.012 to 0.082)	0.051 (0.026 to 0.076)	0.086 (0.052 to 0.12)	0.086 (0.060 to 0.11)
Logged interleukin-6 (pg ml^–1^)^a^	0.043 (0.004 to 0.083)	0.13 (0.006 to 0.020)	0.046 (0.005 to 0.086)	0.026 (0.017 to 0.035)

Abbreviation: CI, confidence interval.

a Total effect: the total effect of maternal education on the cardiovascular risk factor.

b Indirect effect: the effect of maternal education on the cardiovascular risk factor that is mediated through DXA-assessed total fat mass.

*N*=7722 participants with multivariate imputation.

All analyses adjusted for age, height and height squared.

Results are standardised coefficients from simultaneous linear regressions in path analysis, so they estimate the change in s.d. of cardiovascular risk factor associated with a one s.d. increase in maternal education.

## Supplementary Material

Web Tables 1–4

## References

[bib1] Galobardes B Lynch JW Davey Smith G Childhood socioeconomic circumstances and cause-specific mortality in adulthood: systematic review and interpretation Epidemiol Rev 2004 26 7 21 15234944 10.1093/epirev/mxh008

[bib2] Galobardes B Davey Smith G Lynch JW Systematic review of the influence of childhood socioeconomic circumstances on risk for cardiovascular disease in adulthood Ann Epidemiol 2006 16 91 104 16257232 10.1016/j.annepidem.2005.06.053

[bib3] Galobardes B Lynch JW Davey Smith G Is the association between childhood socioeconomic circumstances and cause-specific mortality established? Update of a systematic review J Epidemiol Community Health 2008 62 387 390 18413449 10.1136/jech.2007.065508

[bib4] Agardh EE Ahlbom A Andersson T Efendic S Grill V Hallqvist J Explanations of socioeconomic differences in excess risk of type 2 diabetes in swedish men and women Diabetes Care 2004 27 716 721 14988291 10.2337/diacare.27.3.716

[bib5] Evans JMM Newton RW Ruta DA MacDonald TM Morris AD Socio-economic status, obesity and prevalence of type 1 and type 2 diabetes mellitus Diabet Med 2000 17 478 480 10975218

[bib6] Shrewsbury V Wardle J Socioeconomic status and adiposity in childhood: a systematic review of cross-sectional studies 1990–2005 Obesity 2008 16 275 284 18239633 10.1038/oby.2007.35

[bib7] Owen CG Whincup PH Orfei L Chou Q-A Rudnicka AR Wathern AK Is body mass index before middle age related to coronary heart disease risk in later life? Evidence from observational studies Int J Obes 2009 33 866 877 10.1038/ijo.2009.102 PMC2726133 19506565

[bib8] Davey Smith G Hart C Insulin resistance syndrome and childhood social conditions Lancet 1997 349 284 285 9014935 10.1016/s0140-6736(05)64894-5

[bib9] Lawlor DA Ebrahim S Davey Smith G Socioeconomic position in childhood and adulthood and insulin resistance: cross sectional survey using data from the British women's heart and health study Br Med J 2002 325 805 807 12376440 10.1136/bmj.325.7368.805 PMC128946

[bib10] Lawlor DA Harro M Wedderkopp N Andersen LB Sardinha LB Riddoch CJ The association of socioeconomic position with insulin resistance among children from northern (Denmark), eastern (Estonia) and southern (Portugal) Europe: findings from the European youth heart study Br Med J 2005 331 183 186 16037446 10.1136/bmj.331.7510.183 PMC1179757

[bib11] Loucks EB Magnusson KT Cook S Rehkopf DH Ford ES Berkman LF Socioeconomic position and the metabolic syndrome in early, middle, and late life: evidence from NHANES 1999–2002 Ann Epidemiol 2007 17 782 790 17697786 10.1016/j.annepidem.2007.05.003

[bib12] Batty GD Leon DA Socio-economic position and coronary heart disease risk factors in children and young people Eur J Public Health 2002 12 263 272 12506501 10.1093/eurpub/12.4.263

[bib13] van Lenthe FJ Boreham CA Twisk JW Strain JJ Savage JM Davey Smith G Socio-economic position and coronary heart disease risk factors in youth. Findings from the young hearts project in Northern Ireland Eur J Public Health 2001 11 43 50 11276571 10.1093/eurpub/11.1.43

[bib14] Ness AR Leary S Reilly J Wells J Tobias J Clark E The social patterning of fat and lean mass in a contemporary cohort of children Int J Pediatr Obes 2006 1 59 61 17902216 10.1080/17477160600569339

[bib15] Golding J Pembrey M Jones R the ALSPAC Study Team ALSPAC—The avon longitudinal study of parents and children I. Study methodology Paediatr Perinatal Epidemiol 2001 15 74 87 10.1046/j.1365-3016.2001.00325.x 11237119

[bib16] Morris NM Udry JR Validation of a self-administered instrument to assess stage of adolescent development J Youth Adolesc 1980 9 271 280 24318082 10.1007/BF02088471

[bib17] Duke PM Litt IF Gross RT Adolescents' self-assessment of sexual maturation Pediatrics 1980 66 918 920 7454482

[bib18] Myers GL Kimberly MM Waymack PP Smith SJ Cooper GR Sampson EJ A reference method laboratory network for cholesterol: a model for standardization and improvement of clinical laboratory measurements Clin Chem 2000 46 1762 1772 11067811

[bib19] Wallace AM McMahon AD Packard CJ Kelly A Shepherd J Gaw A Plasma leptin and the risk of cardiovascular disease in the west of Scotland coronary prevention study (WOSCOPS) Circulation 2001 104 3052 3056 11748099 10.1161/hc5001.101061

[bib20] Sergeant JC Firth D Relative index of inequality: definition, estimation, and inference Biostatistics 2006 7 213 224 16192414 10.1093/biostatistics/kxj002

[bib21] Kraemer HC Stice E Kazdin A Offord D Kupfer D How do risk factors work together? Mediators, moderators, and independent, overlapping, and proxy risk factors Am J Psychiatry 2001 158 848 856 11384888 10.1176/appi.ajp.158.6.848

[bib22] Hafeman DM Schwarz S Opening the black box: a motivation for the assessment of mediation Int J Epidemiol 2009 38 838 845 19261660 10.1093/ije/dyn372

[bib23] Avenell A Brown TJ McGee MA Campbell MK Grant AM Broom J What are the long-term benefits of weight reducing diets in adults? A systematic review of randomized controlled trials J Hum Nutr Diet 2004 17 317 335 15250842 10.1111/j.1365-277X.2004.00531.x

[bib24] O'Meara S Riemsma R Shirran L Mather L ter Riet G A systematic review of the clinical effectiveness of orlistat used for the management of obesity Obes Rev 2004 5 51 68 14969507 10.1111/j.1467-789x.2004.00125.x

[bib25] Chanoine JP Hampl S Jensen C Boldrin M Hauptman J Effect of orlistat on weight and body composition in obese adolescents J Am Med Assoc 2005 23 2873 2883 10.1001/jama.293.23.2873 15956632

[bib26] Daniels SR Long B Crow S Styne D Sothern M Vargas-Rodirguez I Cardiovascular effects of sibutramine in the treatment of obese adolescents: results of a randomized, double-blind, placebo-controlled study Pediatrics 2007 120 e147 e157 17576783 10.1542/peds.2006-2137

[bib27] Freathy RM Timpson NJ Lawlor DA Pouta A Ben-Shlomo Y Ruokonen A Common variation in the FTO gene alters diabetes-related metabolic traits to the extent expected given its effect on BMI Diabetes 2008 57 1419 1426 18346983 10.2337/db07-1466 PMC3073395

[bib28] Timpson NJ Harbord R Davey Smith G Zacho J Tybjaerg-Hansen A Nordestgaard BG Does greater adiposity increase blood pressure and hypertension risk? Mendelian randomization using the FTO/MC4R genotype Hypertension 2009 54 84 90 19470880 10.1161/HYPERTENSIONAHA.109.130005

[bib29] Baron RM Kenny DA The Moderator-mediator variable distinction in social psychological research: conceptual, strategic, and statistical considerations J Pers Soc Psychol 1986 51 1173 1182 3806354 10.1037//0022-3514.51.6.1173

[bib30] Wright S The method of path coefficients Ann Math Stat 1934 5 161 215

[bib31] Schumacker RE Lomax RG A beginner's guide to structural equation modeling Lawrence Erlbaum Associates; Mawhah, NJ 2004

[bib32] http://www.statmodel.com/index.shtml [computer program].2007

[bib33] Royston P Multiple imputation of missing values Stata J 2004 4 227 241

[bib34] McClaren L Socioeconomic status and obesity Epidemiol Rev 2007 29 29 48 17478442 10.1093/epirev/mxm001

[bib35] Okasha M McCarron P McEwan J Davey Smith G Childhood social class and adulthood obesity: findings from the Glasgow alumni cohort J Epidemiol Community Health 2003 57 508 509 12821695 10.1136/jech.57.7.508 PMC1732524

[bib36] Davey Smith G McCarron P Okasha M McEwen J Social circumstances in childhood and cardiovascular disease mortality: prospective observational study of Glasgow university students J Epidemiol Community Health 2001 55 340 341 11297656 10.1136/jech.55.5.340 PMC1731885

[bib37] Conen D Glynn RJ Ridker PM Buring JE Albert MA Socioeconomic status, blood pressure progression, and incident hypertension in a prospective cohort of female health professionals Eur Heart J 2009 30 1305 1306 19297384 10.1093/eurheartj/ehp072 PMC2721710

[bib38] Grotto I Huerta M Sharabi Y Hypertension and socioeconomic status Curr Opin Cardiol 2008 23 335 339 18520717 10.1097/HCO.0b013e3283021c70

[bib39] Colhoun HM Hemingway H Poulter NR Socio-economic status and blood pressure: an overview analysis J Hum Hypertens 1998 12 91 110 9504351 10.1038/sj.jhh.1000558

[bib40] Brion MA Ness AR Davey Smith G Leary SD Association between body composition and blood pressure in a contemporary cohort of 9-year-old children J Hum Hypertens 2007 21 283 290 17273154 10.1038/sj.jhh.1002152 PMC2077359

[bib41] Sattar N Williams K Sniderman AD D'Agostino R Haffner SM Comparison of the associations of apolipoprotein B and non-high-density lipoprotein cholesterol with other cardiovascular risk factors in patients with the metabolic syndrome in the insulin resistance atherosclerosis study Circulation 2004 110 2687 2693 15492304 10.1161/01.CIR.0000145660.60487.94

[bib42] Walldius G Jungner I Holme I Aastveit AH Kolar W Steiner E High apolipoprotein B, low apolipoprotein A-I, and improvement in the prediction of fatal myocardial infarction (AMORIS study): a prospective study Lancet 2001 358 2026 2033 11755609 10.1016/S0140-6736(01)07098-2

[bib43] Fairclough SJ Boddy LM Hackett AF Stratton G Associations between children's socioeconomic status, weight status, and sex, with screen-based sedentary behaviours and sport participation Int J Pediatr Obes 2009 4 299 305 19922045 10.3109/17477160902811215

[bib44] Jimenez-Pavon D Kelly J Reilly J Associations between objectively measured habitual physical activity and adiposity in children and adolescents: systematic review Int J Pediatr Obes 2009(e-pub ahead of print). 10.3109/17477160903067601 19562608

[bib45] Ness AR Leary SD Mattocks C Blair SN Reilly JJ Wells J Objectively measured physical activity and fat mass in a large cohort of children Plos Med 2007 4 e97 17388663 10.1371/journal.pmed.0040097 PMC1831734

[bib46] Riddoch CJ Mattocks C Deere K Saunders J Kirkby J Tilling K Objective measurement of levels and patterns of physical activity Arch Dis Child 2007 92 963 969 17855437 10.1136/adc.2006.112136 PMC2083612

[bib47] Rifai N Merrill JR Holly RG Postprandial effect of a high fat meal on plasma lipid, lipoprotein cholesterol and apolipoprotein measurements Ann Clin Biochem 1990 27 489 493 2281930 10.1177/000456329002700512

[bib48] Shand B Elder P Scott R Frampton C Willis J Biovariability of plasma adiponectin Clin Chem Lab Med 2006 44 1264 1268 17032140 10.1515/CCLM.2006.227

[bib49] Poppitt SD Keogh GF Lithander FE Wang Y Mulvey TB Chan YK Postprandial response of adiponectin, interleukin-6, tumor necrosis factor-alpha, and C-reactive protein to a high-fat dietary load Nutrition 2008 24 322 329 18262390 10.1016/j.nut.2007.12.012

[bib50] Emerging Risk Factors Collaboration Di Angelantonio E Sarwar N Perry P Kaptoge S Ray KK Major lipids, apolipoproteins, and risk of vascular disease J Am Med Assoc 2009 302 1993 2000 10.1001/jama.2009.1619 PMC3284229 19903920

