# Supplementary material for: Are there socioeconomic inequalities in cardiovascular risk factors in childhood, and are they mediated by adiposity? Findings from a prospective cohort study
Source: Int J Obes (Lond). 2010 Mar 16;34(7):1149–59. doi: 10.1038/ijo.2010.52 (PMC4052430; doi:10.1038/ijo.2010.52)
Supplement: Web Tables 1–4 [file ijo201052x1.doc]

# Web Tables

**Web Table 1: Comparing children who attended the 10-year follow-up clinic with the full ALSPAC cohort**

|  | **Children attending 10-year follow-up clinic (N=7772)** | **Full ALSPAC cohort (N=14,062)** | **p value** |
| --- | --- | --- | --- |
| Maternal education  Less than O-Level  O-Level  A-Level  Degree or above | 1577 (22.4%)  2476 (35.2%)  1875 (26.6%)  1117 (15.9%) | 3753 (30.4%)  4330 (34.7%)  2803 (22.4%)  1607 (12.9%) | <0.001 |
| Mean maternal age, years (SD) | 29.04 (4.58) | 28.00 (4.96) | <0.001 |
| Mean maternal BMI, kg/m^2^ (SD) | 22.92 (3.75) | 22.93 (3.85) | 0.86 |
| Mean birth weight, kg (SD) | 3.41 (0.55) | 3.38 (0.58) | 0.003 |

## Web Table 2: Inequalities in DXA-assessed total fat mass and cardiovascular risk factors at 9.9 years, quantified by the Slope Index of Inequality (SII) by maternal education. *Non-missing dataset*

|  | **Boys** | | **Girls** | |
| --- | --- | --- | --- | --- |
|  | **SII*** | **(95% Confidence Interval)** | **SII** | **(95% Confidence Interval)** |
| **Inequalities in adiposity** |  |  |  |  |
| ***(difference in geometric means comparing most to least deprived (null value = 1)*** | | | | |
| **Total fat mass**** | 1.14 | (0.99 to 1.30) | 1.37 | (1.23 to 1.54) |
|  |  |  |  |  |
| **Inequalities in cardiovascular risk factors** | | | | |
| ***(difference in means comparing most to least deprived (null value = 0)*** | | | | |
| **Cholesterol (mmol/l)** | 0.090 | (-0.0060 to 0.19) | 0.089 | (-0.012 to 0.19) |
| **Triglycerides (mmol/l)** | -0.013 | (-0.096 to 0.070) | 0.035 | (-0.055 to 0.13) |
| **High density lipoprotein (mmol/l)** | 0.033 | (-0.013 to 0.079) | -0.039 | (-0.086 to 0.0072) |
| **Apolipoprotein A1 (mg/dl)** | 3.26 | (0.25 to 6.26) | -0.40 | (-3.40 to 2.60) |
| **Apolipoprotein B (mg/dl)** | 2.32 | (0.46 to 4.17) | 3.80 | (1.73 to 5.87) |
| **Adiponectin (mg/ml)** | 471.82 | (-315.2 to 1258.9) | -445.77 | (-1301.6 to 410.1) |
| **Systolic blood pressure** | 1.91 | (0.60 to 3.22) | 2.28 | (1.34 to 4.21) |
| **Diastolic blood pressure** | 0.84 | (-0.11 to 1.80) | 1.80 | (0.85 to 2.75) |
| ***(difference in geometric means comparing most to least deprived (null value = 1)*** | | | | |
| **C-reactive protein (mg/l)*** | 1.00 | (0.84 to 1.20) | 1.35 | (1.13 to 1.62) |
| **Leptin (ng/ml)*** | 1.00 | (0.90 to 1.12) | 1.26 | (1.13 to 1.41) |
| **Interleukin 6 (pg/ml)*** | 1.12 | (0.98 to 1.28) | 1.19 | (1.04 to 1.35) |

*SII represents the mean (or geometric mean) difference between the individuals of lowest and highest socioeconomic position on the hypothetical underlying continuous distribution of maternal education

**adjusted for height and height squared

**Web table 3: Geometric mean DXA-assessed fat mass across categories of maternal education at age 9.9 years**

| Maternal education | Boys | | | Girls | | |
| --- | --- | --- | --- | --- | --- | --- |
|  | N | Geometric mean | 95% confidence interval | N | Geometric mean | 95% confidence interval |
|  |  |  |  |  |  |  |
| Below O-Level | 470 | 6403.49 | (5683.04 to 7215.28) | 431 | 10416.12 | (9420.95 to 11516.41) |
| O-Level | 779 | 6338.05 | (5655.60 to 7102.63) | 742 | 9628.84 | (8753.18 to 10592.10) |
| A-Level | 609 | 6611.04 | (5892.95 to 7416.63) | 592 | 9185.89 | (8338.777 to 10119.06) |
| Degree or above | 383 | 5816.35 | (5159.80 to 6556.45) | 371 | 8813.48 | (7957.05 to 9762.08) |
| P value for trend |  |  | 0.07 |  |  | <0.001 |

## Adjusted for age, height, and height squared to give predicted values at age 9.9 years and at the average height of the sample

## Web table 4: Mean levels of cardiovascular risk factors across categories of maternal education at age 9.9 years

| **Risk factor** | Level of Maternal Education | | | | **p value** | | |
| --- | --- | --- | --- | --- | --- | --- | --- |
|  | **< O-Level** | **O-Level** | **A-Level** | **Degree or above** | **(trend)** | | |
| **Boys** | **N=470** | **N=779** | **N=609** | **N=383** |  | | |
| **Associations with outcomes on a continuous scale: means (95% confidence interval)** | | | | | | |  |
| Cholesterol (mmol/l) | 4.25  (4.19 to 4.31) | 4.19  (4.15 to 4.24) | 4.17  (4.12 to 4.23) | 4.18  (4.11 to 4.24) | 0.078 | | |
| Triglycerides (mmol/l) | 1.11  (1.06 to 1.16) | 1.08  (1.04 to 1.12) | 1.12  (1.07 to 1.61) | 1.09  (1.04 to 1.15) | 0.96 | | |
| High density lipoprotein (mmol/l) | 1.43  (1.41 to 1.46) | 1.44  (1.42 to 1.46) | 1.42  (1.39 to 1.44) | 1.41  (1.38 to 1.44) | 0.15 | | |
| Apolipoprotein A1 (mg/dl) | 138.74  (136.91 to 140.58) | 138.42  (136.97 to 139.88) | 136.33  (134.69 to 137.98) | 136.41  (134.46 to 138.46) | 0.022 | | |
| Apolipoprotein B (mg/dl) | 58.71  (57.58 to 59.84) | 56.90  (56.00 to 57.79) | 56.93  (55.92 to 57.95) | 56.69  (55.42 to 57.95) | 0.026 | | |
| Adiponectin (mg/ml) | 12.84  (12.36 to 13.32) | 12.79  (12.41 to 13.17) | 12.79  (12.36 to 13.22) | 12.37  (11.84 to 12.91) | 0.26 | | |
| Systolic blood pressure | 103.31  (102.51 to 104.11) | 102.54  (102.51 to 104.11) | 102.55  (101.83 to 103.27) | 101.60  (100.70 to 102.49) | 0.010 | | |
| Diastolic blood pressure | 57.42  (56.84 to 58.00) | 57.06  (56.60 to 57.52) | 56.92  (56.40 to 57.44) | 56.71  (56.06 to 57.36) | 0.098 | | |
| **Associations with outcomes on a logged continuous scale: geometric means (95% confidence interval)** | | | | | | |  |
| C-reactive protein (mg/l)* | 0.23  (0.21 to 0.26) | 0.21  (0.19 to 0.23) | 0.23  (0.21 to 0.26) | 0.22  (0.19 to 0.25) | 1.00 | | |
| Leptin (ng/ml)* | 4.79  (4.47 to 5.12) | 4.92  (4.66 to 5.19) | 5.02  (4.72 to 5.34) | 4.75  (4.40 to 5.12) | 0.92 | | |
| Interleukin 6 (pg/ml)* | 0.84  (0.78 to 0.91) | 0.76  (0.71 to 0.81) | 0.78  (0.73 to 0.84) | 0.75  (0.69 to 0.82) | 0.15 | | |
| **Girls** | **N=431** | **N=742** | **N=592** | **N=371** |  | | |
| **Associations with outcomes on a continuous scale: means (95% confidence interval)** | | | | | | |  |
| Cholesterol (mmol/l) | 4.38  (4.32 to 4.44) | 4.34  (4.29 to 4.39) | 4.30  (4.25 to 4.36) | 4.31  (4.24 to 4.38) | 0.074 | | |
| Triglycerides (mmol/l) | 1.21  (1.16 to 1.27) | 1.14  (1.10 to 1.19) | 1.18  (1.13 to 1.23) | 1.15  (1.09 to 1.21) | 0.38 | | |
| High density lipoprotein (mmol/l) | 1.34  (1.32 to 1.37) | 1.36  (1.34 to 1.38) | 1.35  (1.33 to 1.38) | 1.39  (1.35 to 1.42) | 0.11 | | |
| Apolipoprotein A1 (mg/dl) | 132.65  (130.79 to 134.50) | 133.28  (131.84 to 134.73) | 132.74  (131.12 to 134.34) | 132.86  (130.83 to 134.89) | 0.95 | | |
| Apolipoprotein B (mg/dl) | 63.15  (61.87 to 64.43) | 62.23  (61.23 to 63.23) | 60.85  (59.74 to 61.97) | 60.33  (58.93 to 61.74) | 0.001 | | |
| Adiponectin (mg/ml) | 13.09  (12.56 to 13.61) | 13.83  (13.42 to 14.24) | 13.34  (12.88 to 13.80) | 13.89  (13.31 to 14.47) | 0.22 | | |
| Systolic blood pressure | 104.00  (103.11 to 104.89) | 102.97  (102.28 to 103.66) | 102.85  (102.08 to 103.63) | 101.48  (100.51 to 102.45) | <0.001 | | |
| Diastolic blood pressure | 58.10  (57.51 to 58.69) | 57.64  (57.18 to 58.10) | 57.44  (56.92 to 57.95) | 56.47  (55.83 to 57.12) | <0.001 | | |
| **Associations with outcomes on a logged continuous scale: geometric means** | | | | | |  | |
| C-reactive protein (mg/l)* | 0.41  (0.36 to 0.45) | 0.35  (0.32 to 0.38) | 0.32  (0.29 to 0.35) | 0.32  (0.28 to 0.36) | 0.002 | | |
| Leptin (ng/ml)* | 8.88  (8.29 to 9.51) | 7.75  (7.35 to 8.18) | 7.42  (6.99 to 7.88) | 7.41  (6.87 to 7.99) | <0.001 | | |
| Interleukin 6 (pg/ml)* | 1.03  (0.95 to 1.12) | 0.98  (0.92 to 1.04) | 0.89  (0.83 to 0.96) | 0.93  (0.85 to 1.01) | 0.017 | | |

**Web Table 5: Association between DXA-assessed total fat mass and cardiovascular risk factors at age 9.9 years.** *Non-missing dataset*

|  | **Boys** | | **Girls** | |
| --- | --- | --- | --- | --- |
|  | **β** | **95% Confidence Interval** | **β** | **(95% Confidence Interval)** |
| Cholesterol (mmol/l) | 0.00002 | 0.00001 to 0.00002 | 0.00002 | 0.00001 to 0.00002 |
| Triglycerides (mmol/l) | 0.00002 | 0.00002 to 0.00003 | 0.00003 | 0.00003 to 0.00004 |
| High density lipoprotein (mmol/l) | -0.00002 | -0.00002 to -0.00001 | -0.00002 | -0.00002 to -0.00002 |
| Apolipoprotein A1 (mg/dl) | -0.0005 | -0.0007 to -0.0003 | -0.0007 | -0.0009 to -0.0005 |
| Apolipoprotein B (mg/dl) | 0.0006 | 0.0005 to 0.0008 | 0.0007 | 0.0005 to 0.0008 |
| Adiponectin (mg/ml) | -0.06 | -0.11 to -0.009 | -0.20 | -0.25 to -0.14 |
| Systolic blood pressure | 0.0007 | 0.0006 to 0.0007 | 0.0007 | 0.0006 to 0.0008 |
| Diastolic blood pressure | 0.0003 | 0.0002 to 0.0003 | 0.0003 | 0.0002 to 0.0004 |
| Logged C-reactive protein (mg/l) | 0.0001 | 0.0001 to 0.0001 | 0.0001 | 0.0001 to 0.0001 |
| Logged Leptin (ng/ml) | 0.0001 | 0.0001 to 0.00013 | 0.0001 | 0.0001 to 0.0001 |
| Logged Interleukin 6 (pg/ml) | 0.00004 | 0.00003 to 0.00005 | 0.00005 | 0.00004 to 0.00006 |

Linear regression coefficients from regressions of total fat mass on the cardiovascular risk factor, adjusted for age, height and height squared. Age, height and height squared were centred on mean values (for boys and girls separately), such that coefficients represent the change in the cardiovascular risk factor for a one kilogram increase in total fat mass at 9.9 years in a child of average height.
